# Severe Acute Respiratory Syndrome Coronavirus 2 (SARS-CoV-2) Encephalitis With Cerebrospinal Fluid Polymerase Chain Reaction (PCR) Positivity and Normal Neuroimaging: A Case Report

**DOI:** 10.7759/cureus.106694

**Published:** 2026-04-09

**Authors:** Gonçalo Dias, Ana I Franco, Miguel G Santos, Catarina Graveto, Ana Araújo

**Affiliations:** 1 Critical Care, Unidade Local de Saúde Região de Leiria, Leiria, PRT; 2 Psychiatry, Unidade Local de Saúde Região de Leiria, Leiria, PRT; 3 Intensive Care Unit, Unidade Local de Saúde Região de Leiria, Leiria, PRT

**Keywords:** cerebrospinal fluid, encephalitis, neuroinvasion, normal neuroimaging, sars-cov-2, viral encephalitis

## Abstract

Neurological manifestations of severe acute respiratory syndrome coronavirus 2 (SARS-CoV-2) are common, yet direct neuroinvasion remains a rare finding. We report the case of a 46-year-old male presenting with acute delirium and behavioral changes in the total absence of respiratory symptoms. While initial neuroimaging and infectious workups were unremarkable, a repeat lumbar puncture detected SARS-CoV-2 RNA in the cerebrospinal fluid (CSF), supporting the diagnosis of SARS-CoV-2-associated encephalitis. The patient experienced a spontaneous recovery, highlighting the importance of cerebrospinal fluid polymerase chain reaction (CSF PCR) testing even when systemic viral loads are undetectable.

## Introduction

Neurological complications occur in approximately half of hospitalized COVID-19 patients [1]. However, it is critical to distinguish between encephalopathy and encephalitis. Encephalopathy, characterized by altered mental status due to systemic factors such as hypoxia, cytokine storm, or metabolic derangement, affects up to 55% of critically ill COVID-19 patients [2,3]. In contrast, true viral encephalitis, defined by direct central nervous system (CNS) inflammation with evidence of viral invasion or intrathecal immune response, is considerably rarer [3-5].

Direct neuroinvasion, indicated by the detection of SARS-CoV-2 RNA in cerebrospinal fluid (CSF), occurs in only 6-7% of cases with neurological manifestations; indeed, most patients with COVID-19 and neurological symptoms show undetectable CSF viral RNA [6-8]. Early in the pandemic, Moriguchi et al. reported one of the first confirmed cases of SARS-CoV-2 meningoencephalitis with viral RNA detected in CSF but not in nasopharyngeal samples [9]. Subsequent reports, including cases without respiratory involvement, have confirmed the capacity for isolated neurotropism [10]. Diagnosis requires an altered mental status lasting ≥24 hours and supportive laboratory or electrophysiological evidence [11]. 

Cases presenting with the "E-MRI" phenotype (encephalitis with normal MRI findings) account for over half of SARS-CoV-2 encephalitis cases and pose significant diagnostic challenges, as traditional neuroimaging may not reveal parenchymal abnormalities despite confirmed CNS infection [7,12]. Approximately 24% of encephalitis cases occur without pneumonia, further complicating recognition [13]. Distinguishing viral encephalitis from autoimmune encephalitis is critical, as the latter may present with similar clinical features but normal CSF PCR and requires different therapeutic approaches [14].

Objective of this report

We present a case that exemplifies the diagnostic complexity of SARS-CoV-2 neuroinvasion occurring in complete isolation from respiratory disease, with delayed CSF detection despite initial negative testing. This case underscores the need for repeat CSF sampling when clinical suspicion persists, even when systemic viral loads are undetectable, and neuroimaging is unremarkable.

## Case presentation

A 46-year-old male with a history of obesity and hypercholesterolemia was admitted following a 24-hour onset of acute delirium, expressive aphasia, and heteroaggressiveness. On admission, he was febrile with a Glasgow Coma Scale (GCS) score of 14 and an NIHSS of 9. Notably, there were no respiratory symptoms, and his oxygen saturation was 98% on room air. Invasive mechanical ventilation was initiated exclusively for airway protection and control of severe psychomotor agitation.

**Figure 1:**
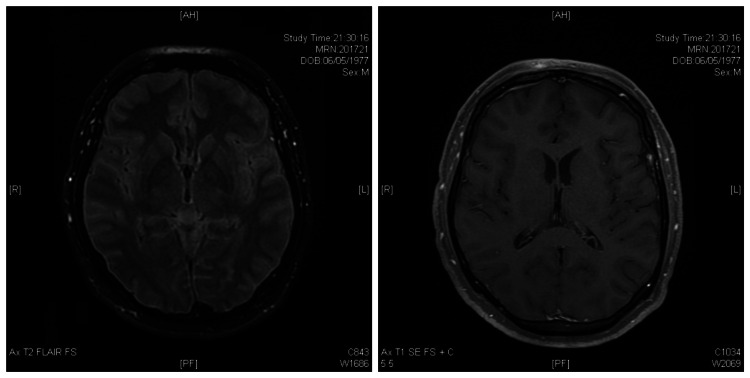
Brain magnetic resonance imaging (FLAIR and post-contrast T1) showing no parenchymal or meningeal abnormalities. Brain magnetic resonance imaging performed on day 0 (Figure 1). (A) Axial T2-weighted fluid-attenuated inversion recovery (FLAIR) with fat suppression demonstrating normal brain parenchyma with no focal signal abnormalities. The absence of cerebrospinal fluid signal suppression is attributed to a technical artifact related to high inspired oxygen fraction during mechanical ventilation and does not reflect true inflammatory or proteinaceous changes. (B) Axial T1-weighted spin-echo with fat suppression following gadolinium administration (T1 SE FS+C) showing no pathological leptomeningeal or parenchymal enhancement. Diffusion-weighted imaging (not shown) demonstrated no areas of restricted diffusion. These findings are consistent with the "E-MRI" phenotype described in SARS-CoV-2 encephalitis, in which a confirmed CNS infection may occur despite normal neuroimaging findings.

Initial blood work showed a C-reactive protein (CRP) of 9.1 mg/L and normal procalcitonin (0.12 ng/mL). CSF analysis (day 0) revealed clear, colorless fluid with normal flow (formal manometry was not performed); laboratory analysis showed neutrophilic pleocytosis (15 cells/µL, 100% PMN) and hyperproteinorrhachia (96 mg/dL). Brain magnetic resonance imaging (MRI) with gadolinium contrast performed under mechanical ventilation showed no parenchymal abnormalities or pathological leptomeningeal enhancement (Figure 1). Respiratory viruses were not included in the initial CSF panel due to the complete absence of pulmonary symptoms, which directed the diagnostic workup toward classic neurotropic pathogens. Empirical treatment with ceftriaxone, vancomycin, acyclovir, and dexamethasone was initiated.

By day 4, despite clinical stability and the absence of fever, the patient’s CRP rose sharply to 115.3 mg/L. This unexplained inflammatory surge, in the absence of a respiratory or urinary focus, prompted clinical reassessment and a repeat lumbar puncture to exclude nosocomial complications or occult pathogens. Given that the initial respiratory SARS-CoV-2 PCR was negative, the virus was not initially suspected. However, it was included in an expanded CSF multiplex PCR panel on day 4 as part of an exhaustive diagnostic workup (Table 1).

**Table 1:** Evolution of hematologic laboratory parameters and cerebrospinal fluid (CSF) findings between admission (day 0) and the fourth day of hospitalization. The early neutrophilic pleocytosis and elevated CSF protein observed in this case are consistent with the initial inflammatory phase of viral CNS infection, which may later evolve toward a lymphocytic predominance. CSF: cerebrospinal fluid; PMN: polymorphonuclear cells; MN: mononuclear cells; CRP: C-reactive protein.

Parameter	Day 0	Day 4	Reference Range (Units)
Blood Analysis			
Serum Leukocytes	10,000	9,700	4,000-10,000 (/μL)
C-Reactive Protein	9.1	115.3	<5.0 (mg/L)
Procalcitonin	0.12	0.12	<0.5 (ng/mL)
Serum Glucose	107	134	74-106 (mg/dL)
CSF Analysis			
CSF Leukocytes	15	12	<5 (/mm³)
Neutrophils	100%	70%	(%)
Lymphocytes	0%	30%	(%)
CSF Protein	96	89	15-45 (mg/dL)
CSF Glucose	70	78	40-70 (mg/dL)

The repeat CSF analysis showed 12 leukocytes/µL (70% PMN) and a protein concentration of 89 mg/dL. While repeat respiratory PCR remained negative, the CSF was positive for SARS-CoV-2 RNA (Table 2). To further differentiate the etiology, an autoimmune encephalitis panel was performed; neural IgG testing for NMDA, LGI1, CASPR2, AMPA1, AMPA2, and GABA-B receptors was negative in both serum and CSF. Having excluded autoimmune and bacterial causes, the diagnosis of SARS-CoV-2 encephalitis was confirmed. The patient experienced spontaneous clinical improvement, completed a 14-day course of antibiotics, and was discharged without neurological deficits.

**Table 2:** Results of the multiplex polymerase chain reaction (PCR) panel in the cerebrospinal fluid (CSF) at admission (day 0) and on the fourth day of hospitalization. The delayed detection of SARS-CoV-2 RNA in CSF despite an initially negative panel highlights the diagnostic value of repeat lumbar puncture when clinical suspicion of encephalitis persists. CSF: cerebrospinal fluid; PCR: polymerase chain reaction; EBV: Epstein-Barr virus.

Microorganism	Day 0 (CSF)	Day 4 (CSF)	Reference Range
Escherichia coli K1	Not detected	Not detected	Not detected
Haemophilus influenzae	Not detected	Not detected	Not detected
Listeria monocytogenes	Not detected	Not detected	Not detected
Neisseria meningitidis	Not detected	Not detected	Not detected
Streptococcus agalactiae	Not detected	Not detected	Not detected
Streptococcus pneumoniae	Not detected	Not detected	Not detected
Cytomegalovirus (CMV)	Not detected	Not detected	Not detected
Enterovirus (EV)	Not detected	Not detected	Not detected
Herpes simplex virus 1 (HSV-1)	Not detected	Not detected	Not detected
Herpes simplex virus 2 (HSV-2)	Not detected	Not detected	Not detected
Human herpesvirus 6 (HHV-6)	Not detected	Not detected	Not detected
Human herpesvirus 7 (HHV-7)	Not tested	Not detected	Not detected
Epstein-Barr virus (EBV)	Not tested	Not detected	Not detected
Human Parechovirus (HPeV)	Not detected	Not detected	Not detected
Varicella-Zoster virus (VZV)	Not detected	Not detected	Not detected
Cryptococcus neoformans/gattii	Not detected	Not detected	Not detected
SARS-CoV-2	Not tested	Detected	Not detected

## Discussion

This case illustrates a rare presentation of SARS-CoV-2-associated encephalitis with evidence of direct neuroinvasion in the total absence of pneumonia, a scenario reported in approximately 24% of encephalitis cases linked to SARS-CoV-2 infection [3,5]. While neurological complications are common in COVID-19, direct viral detection in the CSF is rare [1,6,7], making this a distinct clinical entity from the more common cytokine-mediated encephalopathy [13,15].

A critical teaching point in this case is the potential for diagnostic latency caused by a "respiratory-centric" suspicion. Given the complete absence of respiratory symptoms and negative nasopharyngeal PCR on admission, SARS-CoV-2 was not initially included in the CSF multiplex panel. It was only after clinical reassessment prompted by the unexplained inflammatory surge on day 4 that an expanded panel, including SARS-CoV-2, was performed. Our findings demonstrate that SARS-CoV-2 can exhibit exclusive neurotropism, where the central nervous system (CNS) acts as a primary or sequestered viral reservoir [4]. Clinicians must maintain high vigilance and consider CSF PCR testing even in the absence of systemic viral shedding [5].

The absence of respiratory symptoms and persistently negative nasopharyngeal PCR in our patient suggests direct CNS invasion, bypassing the pharyngeal mucosa. SARS-CoV-2 can enter the nervous system through multiple pathways, including direct neuronal routes, the olfactory nerve (transcribrial route) and the trigeminal nerve, as well as hematogenous routes such as infection of vascular endothelial cells or the "Trojan horse" mechanism via infected leukocytes. Autopsy studies have confirmed SARS-CoV-2 RNA in both the olfactory mucosa and brain, with evidence of viral spread along neuroanatomical structures [16]. Notably, the trigeminal ganglion has been identified as an early and efficient site of viral replication in animal models, from which the virus rapidly disseminates throughout the brain [16]. These direct neural pathways may explain how SARS-CoV-2 can establish compartmentalized CNS infection without detectable respiratory tract involvement, as observed in our case.

The dramatic rise in CRP (from 9.1 to 115.3 mg/L) by day 4, without evidence of secondary bacterial infection, is particularly noteworthy. This disproportionate systemic inflammatory response, in the context of stable procalcitonin (0.12 ng/mL) and negative cultures, likely reflects the spillover of intrathecal cytokine release (IL-6, IL-8, TNF-α) triggered by CNS viral replication, a 'compartmentalized-then-systemic' inflammatory pattern consistent with the cytokine release syndrome described in SARS-CoV-2 encephalitis [17]. Severe COVID-19 neurological disease is characterized by significant systemic upregulation of cytokines and pro-inflammatory mediators associated with elevated acute-phase proteins, including CRP, even in cases where the primary site of infection is the CNS [15,17].

The presence of neutrophilic pleocytosis (15 cells/µL, 100% PMN) in the first 24 hours is consistent with early viral CNS infection. Although lymphocytic pleocytosis is characteristic of viral encephalitis, neutrophils may predominate early in viral infection, with the classical lymphocytic shift typically occurring after 48-72 hours. The persistence of neutrophils at day 4 (70% PMN), with emerging lymphocytes (30%), represents this transitional pattern and does not exclude viral etiology, a finding observed in up to 25% of confirmed viral CNS infections. In West Nile virus encephalitis, for example, 37-45% of patients demonstrate substantial CSF neutrophilia on initial sampling [18].

The negative respiratory SARS-CoV-2 PCR on day 0 followed by positive CSF PCR on day 4 may reflect several mechanisms: viral replication initially confined to the CNS compartment with minimal systemic shedding; viral loads in CSF below detection threshold during early infection; or transient viremia with rapid respiratory clearance but CNS persistence [19]. This temporal dissociation reinforces the concept of isolated neurotropism and underscores the importance of repeated lumbar puncture when clinical suspicion persists.

The "E-MRI-" phenotype observed in this case is consistent with the ENCOVID multicenter study, where more than half of the confirmed encephalitis cases showed no MRI abnormalities despite significant neurological impairment [12]. Gadolinium-enhanced imaging was performed and showed no leptomeningeal or parenchymal enhancement. While meningeal enhancement has been reported in 27-38% of COVID-19 cases with neurological manifestations, its absence does not exclude viral encephalitis, particularly in SARS-CoV-2 infection, where normal neuroimaging is documented in over half of confirmed cases [20]. Furthermore, the systematic exclusion of autoimmune encephalitis was paramount. By confirming the absence of neural antibodies, we reinforced that the neurological dysfunction was a direct result of viral presence rather than a post-infectious autoimmune phenomenon [14,21].

A note on antibiotic management: The 14-day antibiotic course was maintained despite confirmed viral etiology due to several factors reflecting real-world clinical constraints. Empirical broad-spectrum coverage was initiated on day 0, given the acute presentation with neutrophilic pleocytosis, which does not reliably distinguish bacterial from viral meningitis in the first 24-48 hours. A practical limitation in our district hospital setting is that microbiological sample processing is not available seven days per week; definitive CSF PCR results were only available on days 6-7, by which point nearly one week of antibiotics had been administered. The unexplained CRP surge on day 4 further raised concern for possible bacterial superinfection. Given the patient's clinical stability and the low risk of short-term antibiotic continuation, the treating team opted for a conservative approach. In retrospect, earlier de-escalation would have been appropriate once viral etiology was confirmed, highlighting the common tension between diagnostic certainty and therapeutic decision-making in resource-limited settings.

This case has several limitations that warrant acknowledgment. First, we cannot definitively exclude transient systemic SARS-CoV-2 infection prior to presentation, as the patient was not tested before symptom onset. Second, quantitative PCR data (Ct values) were not available, precluding assessment of viral replication dynamics in the CSF. Third, common respiratory coronaviruses and adenoviruses were not tested in the initial CSF panel, as the absence of respiratory symptoms lowered clinical suspicion for these pathogens. However, these viruses were subsequently tested in both CSF (day 4) and respiratory secretions (day 6) and were negative (Table 3), making their contribution to the clinical picture unlikely. Finally, as a single case report, these findings are hypothesis-generating and should not be interpreted as practice-changing; further systematic studies are needed to validate the diagnostic approach suggested herein.

**Table 3:** Comparison of respiratory pathogen panel results between CSF (day 4) and respiratory secretions (day 6). The discordance between CSF positivity for SARS-CoV-2 and negative respiratory testing supports the concept of isolated viral neurotropism in this case. CSF: cerebrospinal fluid; PCR: polymerase chain reaction; MERS-CoV: Middle East Respiratory Syndrome Coronavirus.

Microorganism	Day 4: CSF	Day 6 - Respiratory Secretions	Reference Range
Adenovirus	Not detected	Not detected	Not detected
Coronavirus (229E, HKU1, NL63, OC43)	Not detected	Not detected	Not detected
MERS-CoV	Not detected	Not detected	Not detected
SARS-CoV-2	Detected	Not detected	Not detected
Metapneumovirus	Not detected	Not detected	Not detected
Rhinovirus/Enterovirus	Not detected	Not detected	Not detected
Influenza (A, A/H3, B)	Not detected	Not detected	Not detected
Parainfluenza (1, 2, 3, 4)	Not detected	Not detected	Not detected
Respiratory Syncytial Virus	Not detected	Not detected	Not detected
Bordetella pertussis/parapertussis	Not detected	Not detected	Not detected
Chlamydia pneumoniae	Not detected	Not detected	Not detected
Mycoplasma pneumoniae	Not detected	Not detected	Not detected

Key diagnostic cues for SARS-CoV-2 encephalitis without respiratory involvement

Clinicians should consider SARS-CoV-2 encephalitis when the following constellation of findings is present: (1) acute neuropsychiatric symptoms (delirium, behavioral changes, altered consciousness) without respiratory complaints; (2) early neutrophilic CSF pleocytosis evolving toward a lymphocytic profile; (3) elevated CSF protein with normal or mildly elevated glucose; (4) disproportionate systemic inflammatory response (rising CRP) without identifiable infectious focus; (5) normal or non-specific brain MRI findings (E-MRI phenotype); and (6) negative initial respiratory PCR, which should not exclude the diagnosis. In such cases, repeat lumbar puncture with expanded viral PCR testing, including SARS-CoV-2, is warranted even when systemic viral shedding is undetectable.

In conclusion, this case supports the role of repeated and expanded CSF analysis when clinical suspicion of CNS infection persists [6,7]. The delayed detection in the CSF highlights that viral loads in the CNS may be transient or initially below the detection threshold, justifying a low threshold for repeat lumbar puncture [6,7].

## Conclusions

This case supports the hypothesis that SARS-CoV-2 encephalitis can manifest as a primary neurological syndrome without respiratory involvement and that direct neuroinvasion may occur independently of detectable systemic infection. Red flags that should prompt consideration of this diagnosis include acute neuropsychiatric symptoms with disproportionate systemic inflammation, neutrophilic CSF pleocytosis, and normal neuroimaging, particularly when initial respiratory PCR is negative. Physicians should maintain a high index of suspicion and consider repeat CSF PCR testing, as direct evidence of neuroinvasion may be transient or delayed. While these observations are derived from a single case and require validation in larger cohorts, they highlight the importance of maintaining diagnostic vigilance for SARS-CoV-2 neuroinvasion even in the absence of respiratory disease.
